# G-FORCE: the effectiveness of group psychotherapy for Cluster-C personality disorders: protocol of a pragmatic RCT comparing psychodynamic and two forms of schema group therapy

**DOI:** 10.1186/s13063-023-07309-w

**Published:** 2023-04-29

**Authors:** Birre B. van den Heuvel, Jack J. M. Dekker, M. Daniëls, Henricus L. Van, Jaap Peen, Judith Bosmans, Arnoud Arntz, Marcus J. H. Huibers

**Affiliations:** 1 Arkin Mental Health Care, Amsterdam, the Netherlands; 2 Vrije Universiteit, Amsterdam, the Netherlands; 3 Department of Health Sciences, Faculty of Science, Vrije Universiteit Amsterdam, Amsterdam Public Health Research Institute, Amsterdam, the Netherlands; 4 Universiteit Van Amsterdam, Amsterdam, the Netherlands; 5 Department of Clinical Psychology, Utrecht University, Utrecht, the Netherlands

**Keywords:** Personality disorders, Group psychotherapy, Randomized controlled trial, Pragmatic trial, Schema therapy, Group schema therapy, Psychodynamic group therapy, Working mechanisms, Cluster-C

## Abstract

**Background:**

Cluster-C personality disorders (PDs), characterized by a high level of fear and anxiety, are related to high levels of distress, societal dysfunctioning and chronicity of various mental health disorders. Evidence for the optimal treatment is extremely scarce. Nevertheless, the need to treat these patients is eminent. In clinical practice, group therapy is one of the frequently offered approaches, with two important frameworks: schema therapy and psychodynamic therapy. These two frameworks suggest different mechanisms of change, but until now, this has not yet been explored. The purpose of the present G-FORCE trial is to find evidence on the differential (cost)effectiveness of two forms of schema group therapy and psychodynamic group therapy in the routine clinical setting of an outpatient clinic and to investigate the underlying working mechanisms and predictors of outcome of these therapies.

**Methods:**

In this mono-centre pragmatic randomized clinical trial, 290 patients with Cluster-C PDs or other specified PD with predominantly Cluster-C traits, will be randomized to one of three treatment conditions: group schema therapy for Cluster-C (GST-C, 1 year), schema-focused group therapy (SFGT, 1.5 year) or psychodynamic group therapy (PG, 2 years). Randomization will be pre-stratified on the type of PD. Change in severity of PD (APD-IV) over 24 months will be the primary outcome measure. Secondary outcome measures are personality functioning, psychiatric symptoms and quality of life. Potential predictors and mediators are selected and measured repeatedly. Also, a cost-effectiveness study will be performed, primarily based on a societal perspective, using both clinical effects and quality-adjusted life years. The time-points of assessment are at baseline, start of treatment and after 1, 3, 6, 9, 12, 18, 24 and 36 months.

**Discussion:**

This study is designed to evaluate the effectiveness and cost-effectiveness of three formats of group psychotherapy for Cluster-C PDs. Additionally, predictors, procedure and process variables are analysed to investigate the working mechanisms of the therapies. This is the first large RCT on group therapy for Cluster-C PDs and will contribute improving the care of this neglected patient group. The absence of a control group can be considered as a limitation.

**Trial registration:**

CCMO, NL72826.029.20. Registered on 31 August 2020, first participant included on 18 October 2020.

## Administrative information

**Table:** 

Title {1}	**G-FORCE: The Effectiveness of Group Psychotherapy for Cluster-C Personality Disorders: A pragmatic RCT comparing Psychodynamic and Two Forms of Schema Group Therapy**
Trial registration {2a and 2b}.	NL72826.029.20 [Registry ID: CCMO]. Registered on 20–08-31 ICTRP: NL 8607 [International Clinical Trial Platform] 2020–05-11
Protocol version {3}	Version 2. 20 February 2023,
Funding {4}	This research is funded by Arkin.
Author details {5a}	B. van den Heuvel, MSc: Arkin Mental Health Care, Amsterdam, the Netherlands Prof. J.J.M. Dekker, PhD: Department of Clinical Psychology, Vrije Universiteit Amsterdam, the Netherlands M. Daniëls, MSc: Arkin Mental Health Care, Amsterdam, the Netherlands H.L. Van, PhD: Arkin Mental Health Care, Amsterdam, the Netherlands J. Peen, PhD: Arkin Mental Health Care, Amsterdam, the Netherlands Prof. J.E. Bosmans, PhD: Department of Health Sciences, Faculty of Science, Vrije Universiteit Amsterdam;Amsterdam Public Health research institute, The Netherlands Prof. A. Arntz, PhD: Department of Clinical Psychology, Universiteit van Amsterdam, the Netherlands Prof. M.J. H. Huibers, PhD: Utrecht University, Department of Clinical Psychology, and Arkin Mental Health Care, Amsterdam, the Netherlands
Name and contact information for the trial sponsor {5b}	H.L. Van: Arkin Mental Health Care, Domselaerstraat 128, 1093 MB, Amsterdam, the Netherlands, rien.van@arkin.nl
Role of sponsor {5c}	The sponsor (Arkin) had no role in the design of this study and will not have any role during its execution, analyses, interpretation of the data, or decisions about submitting results

## Introduction

### Background and rationale {6a}

Cluster-C PDs are the most prevalent PDs, with a prevalence rate of 3–9% in the general population [87] and in specialized mental health care up to 54% [2]. Cluster-C PDs are characterized by a high level of fear and anxiety and comprise of the classifications of avoidant, obsessive–compulsive and dependent personality disorder. These PDs are related to various other common mental health disorders [65, 69] and severe impairment of quality of life and societal functioning [80]. Many of these patients receive generally available treatments such as psychodynamic or cognitive behavioural therapy, sometimes specifically adapted on Cluster-C related pathology. However, evidence for effectiveness of these treatments is scarce, partly because research in PDs has predominantly focused on borderline PD, largely neglecting Cluster-C pathology [47, 53]. Only a few randomized controlled trials on treatment for Cluster-C PDs have been conducted [53]. Due to this lack of well-powered controlled effectiveness studies, until now, no international guidelines for treating Cluster-C PDs exist [7].

Nevertheless, in daily clinical practice, the need to treat these patients is eminent. Group therapy is one of the frequently offered approaches in daily practice, in part because it is assumed to have many advantages for patients with Cluster-C pathology. The group can function as a ‘micro cosmos’ where problematic interactional problems that are central to personality pathology will surface, making it possible to address and change these patterns on the spot [103]. Avoidant or controlling coping mechanisms and attitudes, typical for Cluster-C PDs, could be especially challenged within and through the context of interactions during a group treatment.

Alongside the current study, a second RCT is running at the same mental health care centre on the effectiveness of individual treatments of Cluster-C PDs: I-FORCE [22]. G-FORCE and I-FORCE have similar procedures and make use of identical measurements and time-points of assessment. In this protocol article, it will be explicitly marked when descriptions of the research process are similar or identical to the description in the protocol paper of I-FORCE.

### Explanation for the choice of comparators {6b}

#### Psychotherapies

In the field of schema therapy, various group formats have been developed. Schema therapy integrates cognitive behaviour therapy and concepts and techniques from attachment theory, experiential and psychodynamic therapy [104]. Schema therapy aims to restore dysfunctional schema’s, i.e. representations of the self that stem from early childhood, and to change schema modes to more healthy forms. An internationally frequently applied schema group format is group schema therapy (GST), as developed by Farrell and Shaw [30], which has demonstrated its effectiveness for treating borderline PD [31, 100]. Recently, GST was adapted to address Cluster-C pathology [86]. Preliminary observations from an ongoing RCT on patients with avoidant PD and Social Anxiety Disorder [8] suggests it is feasible and effective for this patient group. In another running multi-centre RCT, GST-C is compared with individual schema therapy and TAU [43].

Another type of schema group therapy that is frequently offered for Cluster-C PDs in the Netherlands is schema-focused group therapy (SFGT), developed by Aalders and van Dijk [1]. This group format combines a highly structured, schema-focused part with an unstructured, dynamic approach, characterized by an open dialogue and group dynamic interventions. Based on a naturalistic cohort study, this format seems to be effective with medium effect sizes for a broad group of patients with mixed personality disorders [55].

For decades, psychodynamic group therapy (PG) is another widely offered treatment for Cluster-C patients. ‘Psychodynamic group therapy’ is an umbrella term for a variety of approaches ranging from group analysis [33] to interpersonal group therapy [102]. PG combines psychodynamic and group dynamic theories and interventions and is characterized by an open group dialogue, allowing the therapist to be maximally responsive to relevant interactions and permissive to discuss less conscious phenomena that could be derived from verbal and nonverbal expressions of participants or from the group process. A systematic review of mainly open cohort studies [14] provides some support for the effectiveness of PG in general for a mixed diagnostic group of patients.

We are aware of only one RCT on psychodynamic group therapy [63, 35] in which the effectiveness of 80 sessions was compared to a variant of 20 sessions in a heterogeneous patient sample. Of the participants, 45% were diagnosed with a PD, mostly Cluster-C. For the PD patients, the 80 sessions group therapy resulted in a better outcome on symptom level and interpersonal functioning with a medium effect size, which was even maintained until after 7 years of follow-up.

The choice of treatment modalities in this study constitutes a continuum with the highly structured, protocolized, shorter GST-C on one end, and the highly unstructured, longer PG on the other end. SFGT takes a mid-position on this continuum, with a structured schema focused part, and an unstructured group dynamic part. The choice for these three group therapies is rooted in pragmatic reasons: in this pragmatic clinical trial, we wanted to research existing group treatments that are provided in the mental health care system of the Netherlands. Moreover, this continuum of treatment modalities gives the opportunity to investigate the effect of amount of structure and room for group dynamics and the difference in duration and to explore who benefits the most from which type of group therapy.

The current study will compare the three aforementioned forms of group therapy, that differ in therapeutic approach and duration in a sample of Cluster-C patients: GST-C, SFGT and PG.

#### Potential mediators

In order to understand why, how and for whom a therapy works, it is useful to explore predictors and mechanisms of change in group psychotherapy for PDs, which could be potentially helpful in adapting and optimizing interventions [21].

In therapies for PDs, general mechanisms of change such as therapeutic alliance and emotional processing appeared to be associated with outcome [52, 36]. Of these, the therapeutic alliance is the most frequently demonstrated and robust common factor of change [37, 36].

However, in group therapy, the alliance with the group as whole and with its members is supposed to be just as important as the alliance with the therapist. This is referred to as *group cohesion.* In a meta-analysis by Burlingame et al. [18], cohesion in group therapy indeed appeared to be associated with outcome, across different forms of group therapy and diagnoses. The effect appeared to be related to an interpersonal approach in the group, a group size of 5–9 participants and a duration of more than 12 sessions. However, due to the correlational character of the included studies, a causal relationship could not be established, nor a potential influence of the theoretical approach of the group therapy or diagnoses of the patients [18].

In addition to general working mechanisms, specific mechanisms of change are derived from the theoretical frameworks. For schema therapy, the presumed working mechanism is the change in schema modes. Two schema modes in particular, the Healthy Adult (integrated, healthy functioning state) and the Vulnerable Child (state of anxiety, sadness, loneliness or abuse/neglect), seem to be pivotal in improving personality functioning [101]. In psychodynamic therapies, increase in insight or self-understanding and affect awareness are hypothesized to be distinctive mechanisms of change [42, 46]. Amelioration of self-image, expression of affect and change of defence style are theoretically considered to be specific psychodynamic working mechanisms as well [91].

Adequate research on mechanisms of change needs a demonstration of a statistical association and a temporal relationship between treatment intervention, mediator and outcome. Consistency and specificity of the mediator also needs to be proven [51]. Hence, the measurement of potential mediators and outcomes at multiple points during and after therapy and across treatment conditions is a prerequisite [58]. In this study, these recommendations will be followed. Additionally, to explore the relationship between certain interventions, mediators and outcome, treatment interventions will be specified by a treatment interventions list. After each group therapy session, the therapist indicates which of the core interventions per treatment was applied. On this list, core interventions of the specific treatment modalities (schema therapeutic or psychodynamic) and general group dynamic interventions are described.

#### Potential predictors/moderators

Potential predictors and moderators will be studied to answer the question *which* therapy works for *whom*. Apart from general predictors (sociodemographic variables, subtype of personality disorder, severity of psychiatric symptoms, severity of the personality disorder), the following specific potential predictors are selected, based on the outcomes of an expert meeting of experienced professionals on the treatment of Cluster-C PDs. First, childhood trauma is assumed to underlie to some extent the development of personality pathology, and adequately processing these events will contribute to effective treatment [5, 6]. For various psychiatric disorders, childhood trauma has been found to have a negative impact on treatment response and outcome [68, 19].

Second, because of the high comorbidity between autism spectrum disorder and Cluster A and C personality disorders, and reduced effectiveness of treatment of affective disorders for patients with autism [96, 64, 99], the predictive value of autism traits is investigated.

Third, personality organization as defined by Kernberg [54] is chosen as a potential predictor. Level of personality organization is specified by the assessment of three domains: identity, primitive defence mechanisms and reality testing, covering a continuum ranging from neurotic personality organization to borderline personality organization to psychotic personality organization, with increasing severity of disturbance. Higher levels of personality organization were associated with better treatment outcome [57]. The impact of the level of personality organization on the outcome of treatment for Cluster-C PDs has never been studied before.

Fourth, vulnerable narcissism has found to be specifically manifest in avoidant and obsessive–compulsive PD [27, 67, 72] and is strongly related to anxious and avoidant attachment; negative irrational beliefs about the self, world and future; and maladaptive and avoidant coping strategies [50]. Because of these associations, a possible negative influence on treatment outcome might be expected.

### Objectives {7}

The aim of this pragmatic randomized clinical trial is to compare the effectiveness and cost-effectiveness of three group therapy formats that are regularly delivered in clinical practice to patients with a Cluster-C PD in a superiority study design. The primary outcome is the severity of the Cluster C-PD. The slopes over time of the three treatments (GST-C, SFGT and PG) from baseline to 24 months will be compared. Because of the variable durations of the treatments, we will make a comparison over a time window of 24 months, with assessments taking place at baseline, 3, 6 and 9 months, and at the different end points of the treatments at 12, 18 and 24 months, respectively. Next to the a priori tests over 24 months, we will explore differences between the outcomes of the three treatments on the slopes over time between start of treatment and the respective (different) end points. Differences in outcome at 36 months follow-up will be studied separately. No hypothesis was formulated about the direction of the difference.

The second aim is to study potential mechanisms of change both across therapies and for each specific theoretical framework by determining mediators in repeated measurements during and after therapy, across treatment conditions [58]. Group cohesion, group climate and working alliance are chosen as hypothesized general working mechanisms for group therapy. Another focus of research is the relationship between specific treatment interventions, mediators and outcome. The hypothesis is that certain interventions will be stronger connected to mediators and outcome than others. For example, we expect group dynamic interventions to be positively related to group cohesion.

The third aim is to study potential predictors and its moderating impact on outcome. The hypothesis is that for patients with higher levels of childhood trauma, schema therapy will be more effective, because of its use of trauma-oriented techniques, such as imaginary rescripting [34, 74]. Another hypothesis is that a more structured therapy such as GST-C will yield better results for patients with more severe personality pathology, e.g. borderline personality organization [54]. These patients are less able to tolerate high anxiety levels which can be provoked by the unstructured and exploratory character of PG [39]. Autism traits and vulnerable narcissism will be studied exploratively.

With this study, we hope to strengthen the evidence of the effectiveness of group therapy for Cluster-C PDs and to contribute to answer on the question *which* therapy works *for whom* and *why exactly.* This will enable us to recommend a specific therapy to subgroups of patients with the best chance of being effective for an individual patient. This approach may improve the overall effectiveness of group therapy by a strengthened focus on its working mechanisms.

### Trial design {8}

The G-FORCE study is a randomized controlled clinical trial with three parallel groups: GST-C, SFGT and PG. The patient allocation ratio is 1:1:1. G-FORCE is a pragmatic ecologically valid RCT, in which broad inclusion criteria (among which the permission of comorbidity and use of psychopharmacological medication), the routine clinical setting and the guaranteed clinical equipoise of the three treatments are important features [34]. This means that patients with a Cluster-C PD who are characterized by a high amount and a wide range of psychiatric comorbidity will only be excluded in case of acute need for (intensive) treatment.

## Methods: participants, interventions and outcomes

### Study setting {9}

The study is performed at the NPI, a Dutch mental health care centre specialized in PDs (part of Arkin Mental Health Care). The NPI is a tertiary, specialized referral centre for PD treatment with four locations in and around Amsterdam. It is recognized as a TOP Clinical Mental Health Care Institute by the Foundation of TOP Clinical Mental Health Care in the Netherlands. The institute has a long history and expertise in schema therapy and psychodynamic treatments, in group and individual setting, and offers clinical internships for psychiatrists, clinical psychologists and psychotherapists in training. Patients are considered for inclusion if they meet the criteria as defined below.

### Eligibility criteria {10}^1^

#### Inclusion criteria

- Primary diagnosis DSM-5 diagnosis of a Cluster-C PD or otherwise specified PD with predominantly Cluster-C traits (operationalized as a minimum of 5 Cluster-C traits).
- Age 18–65 years
- The willingness and ability to participate in a group treatment of 1–2 years.
- A written informed consent.

#### Exclusion criteria

- (Sub threshold) Cluster A or B PD
- Non-Dutch speaker/reader
- Immediate intensive treatment or hospitalization is needed, e.g. acute suicidality
- Severe psychiatric disorder requiring priority in treatment (autism spectrum disorder; psychotic symptoms; bipolar disorder; severe substance use disorder)
- No fixed home address
- Estimated IQ < 80
- Pregnancy or other practical reasons why trial demands cannot be met

#### Therapists

Therapists who are performing the interventions have an official registration (according to the Dutch Individual Healthcare Professions Act) as a health care psychologist, psychotherapist, clinical psychologist or psychiatrist, or are in training for these official registrations. Therapists are trained in the protocols specific for the treatment modalities.

### Interventions

#### Intervention description {11a}

GST-C is based on the protocol of Farrell and Shaw [30] that has been adapted for the Cluster-C patient [86]. The protocol consists of 30 weekly sessions of 90 min, combined with facultative individual sessions with a maximum of 300 min in total, followed by 4 monthly group booster sessions of 60 min each. Change of schema modes with experiential techniques constitutes the core of the GST-C. The structure of the group is semi-open, with new participants enrolling every 10 sessions, with a maximum of 9 participants in total. The group is led by two therapists, with a third therapist appointed to step in if one of the two therapist is absent. Therapists both have an additional minimum registration of junior schema therapist and are trained in group schema therapy according to Farrell and Shaw [30].

SFGT combines a structured schema-focused approach with an open, unstructured part in one session, thereby giving more room to group dynamics than traditional schema therapy groups [1]. This format is developed to be especially suited for patients with a Cluster-C PD, enabling a focus on intrapersonal and interpersonal dynamics. SFGT consists of three phases, with every 20 sessions new participants enrolling in the group and others terminating. Participants commit to a minimum of 40 sessions, with a maximum of 60. Every session starts with 60 min structured schema therapy interventions, mainly experiential exercises aimed at schema mode change. This is followed by an open group dialogue of 45 min in which group dynamic interventions play a central role using, if appropriate, schema language to interpret and communicate. The group is led by a head therapist and a co-therapist. The head therapist has a minimum registration of junior schema therapist.

PG makes use of psychodynamic theories and interventions in combination with a focus on the group dynamics [10]. The sessions have an open dialogue character that facilitates a group process in which personality patterns emerge. This enables the therapist to intervene on both the individual level of the patient and on the interaction between patients and the group as a whole. Typical for the psychodynamic framework is to facilitate insight by relating emotions in the here and now to earlier experiences during life [39] and to interpret the transference with the group therapists and the relation with group members as repetitions of earlier relational experiences [10, 62]. In addition, frequently occurring defence and resistance phenomena are discussed to improve the group process, thus making underlying or avoided feelings or fantasies more conscious and beneficial for the patients [10]. PG gives room to spontaneous interactions between group members, facilitating the possibility of interpersonal learning. Through feedback of the group members and group leaders and self-observation, the increase in insight and self-understanding, change in self-image, activation of affects and change of defence style is facilitated. The duration is 80 sessions (2 years) with 90 min sessions. A new participant enrols when another terminates, with a maximum of 9 participants. The group is led by two group therapists, a head therapist and a co-therapist. The minimum qualification for the head therapist is an official specialisation in group therapy. All therapists are trained in a PG protocol that describes the theoretical framework, process and interventions [90], to increase allegiance of therapists and testing adherence.

#### Criteria for discontinuing or modifying allocated interventions {11b}

If participants decide to discontinue with the study for any reason, they are allowed to finish their current treatment. Hence, a study dropout does not implicate a treatment dropout.

It is also possible that the therapist or the investigator decides to withdraw the patients from the study for medical reasons. Treatment dropout or push out can occur for the following reasons: another severe psychiatric disorder requires priority in treatment, the disorder deteriorates, practical reasons, motivational or commitment problems. Switching between different treatments in the trial is not possible. In case of a need for treatment after dropout, patients will be offered a regular treatment or will be referred to another institution.

#### Strategies to improve adherence to interventions {11c}

For improving adherence to the intervention protocols, all therapists are attending peer supervision meetings with a minimum frequency of every 4 weeks. All groups are discussed at least once a year during the study period. In every peer supervision group one therapist is appointed as adherence attendant. Regular meetings between the adherence attendants and researchers take place. To assess treatment adherence and competence, treatment sessions will be audio recorded. A random sample of 20% of the treatment session recordings will be rated by independent trained judges blind for condition. For GST-C, an existing adherence and competence measure will be used, the Group Schema Therapy Integrity Scale (GST-TI; see [43] and expanded. For SFGT, a new rating scale is developed, based on the Group Schema Therapy Rating Scale-Revised (GSTRS-R, [105]. For PG, no validated adherence and competence scale does exist and will be developed. In addition, relevant deviations from the protocol have to be reported and if necessary, discussed in the G-FORCE research committee, consisting of the coordinating researcher, a clinical expert from every treatment modality, a psychiatrist/clinical researcher and a research assistant.

#### Relevant concomitant care permitted or prohibited during the trial {11d}

The use of psychopharmaca during the group therapy is permitted. In order to strengthen the clinical validity of the trial and to provide good clinical care in case of comorbidity, a limited amount (maximum 10 sessions) of the following psychotherapeutic co-interventions is allowed: EMDR, family or couple therapy or relapse prevention for substance abuse. The decision of adding a co-intervention must be made by the research committee and is monitored. Sensitivity analyses will be performed with these interventions as a covariate. Extra health care costs are incorporated in the cost-effectiveness analysis.

#### Provisions for post-trial care {30}

After termination of the treatment, a period of 6 months without psychotherapy is strongly advised. If for any reason additional care is necessary before these 6 months, the patient can reapply for therapy at the NPI, with a referral by the GP. In this case of reapplication, the research committee must be informed and can be involved in the decision making about the following therapy. If the patient reapplies after 6 months, but before the follow-up measurements are conducted, the same procedure is applicable.

No risk of harm is expected by participating in this trial.

### Outcomes {12}^2^

Baseline assessment takes place right after informed consent and before randomization and start of treatment. Assessments consist of digital questionnaires and semi-structured interviews and are all administered in Dutch. Assessors are trained and will be supervised to ensure quality of data collection of semi-structured interviews. Table 1 shows an overview of the instruments per assessment point.

**Table 1:**
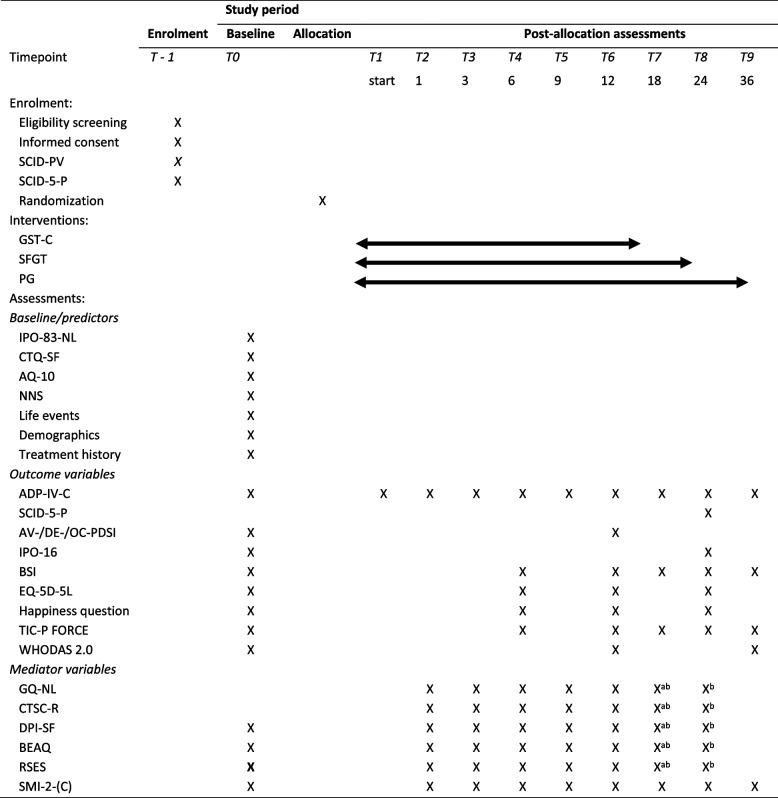
Overview of study design, measurements, and time of assessment

^a^SFGT

^b^PG

#### Primary outcome

The primary outcome is severity of Cluster-C personality pathology measured by the Assessment of DSM-IV Personality Disorders questionnaire (ADP-IV*,* [79]. With this self-report questionnaire, DSM-IV PD criteria are assessed, but because in DSM-5 no important changes are made in the description of PDs, the ADP-IV is still applicable. Patients indicate on a 7-point Likert scale to what degree PD criteria hold for them, ranging from 1 (‘not at all’) to 7 (‘completely’), and whether they experience distress from it (on a range from 1-not at all to 3-definitely). Item construction of the ADP-IV allows for both dimensional and categorical diagnostic evaluation [79]. Adequate internal consistency, validity and reliability were shown consistently in previous studies [28, 78]. A selection of the Cluster-C items from the ADP-IV is made for the primary outcome measure of this trial.

#### Secondary outcomes

##### Personality functioning

With the aim of capturing the complexity of personality functioning, this concept is measured in three ways. First, to explore the predictive value of amount of personality traits (Cluster-C and comorbid PD traits) and to measure remission rates of the Cluster-C personality disorder, the Structural Clinical Interview for DSM-5 personality disorders (SCID-5-P-NL, [4] is used*.* The Dutch version of the SCID-5-P is used for diagnosing PDs at assessment and at 24 months. The exact reliability and validity of the SCID-5-P is unknown, but research has shown that both the original SCID-II and the Dutch version and translations of the SCID-5-P in other languages have adequate to good interrater reliability and test–retest interrater reliability [41, 59, 66, 81] Assessment using the SCID-5-P will be guided by items previously affirmed by the patient on the Structured Clinical Interview for DSM-5 Personality Questionnaire (SCID-5-PV), a self-report questionnaire screening for PDs that will be completed at intake. Items not affirmed on the SCID-5-PV will be assumed to be true negatives; however, if a clinician has reason to believe these are false negatives, such items will be assessed. This method is in accordance with instructions for using the SCID-5-P and enables the assessment of PD symptoms to be based upon self-report combined with a structured clinical interview.

Secondly, the severity of the primary Cluster-C PD is measured with the Avoidant Personality Disorder Severity Index (AVPDSI), Dependent Personality Disorder Severity Index (DEPDSI) or the Compulsive Personality Disorder Severity Index (OCPDSI), the choice depending on what the primary PD is. These semi-structured interviews are developed to assess the frequency and severity of manifestations of the DSM-5 criteria of avoidant, dependent and compulsive PDs. The recall period for these instruments is 3 months. For patients with a main diagnosis 'otherwise specified PD' with predominantly Cluster-C traits, a personalized selection of the Cluster-C traits derived from the AVPDSI, OCPDSI and DEPDSI is made. Psychometric qualities of these instruments are still under research, but excellent interrater agreement (ICC > 0.90) and internal consistency (Cronbach’s *a* > 0.90) is established [43]. Total scores of the AVPDSI, DEPDSI and OCPDSI consist of a total sum of the average symptom scores per subsection of the interview, an average burden score and an average impact score. The scores on the instruments are converted into one severity score by standardizing the raw scores (see [43]).

To ensure quality of data collection of this developed instruments, assessors will be trained and supervised, and all measurements will be audiotaped.

Third, the change in global severity of personality pathology according to Kernberg’s object-relationship framework is measured by the Inventory of Personality Organisation Short Form (IPO-16-NL, [48]. The IPO-16-NL is the Dutch short version of the IPO-83 [20]. Norm scores of the German version are available and psychometric evaluation has shown good internal consistency, reliability, validity and confirmed a one factor structure of general personality dysfunction [106, 107]. The total score on the 16 items represents a dimensional measure of global severity of personality pathology.

##### Psychiatric symptoms

The Brief Symptom Inventory (BSI, [23] is a 53-item self-report instrument that will be used to measure general psychological distress. The answers are scored on a 5-point Likert scale. It is derived from the SCL-90-R and has demonstrated it to be an acceptable short alternative of its longer version [25].

##### Quality of life, happiness, and psychosocial functioning

Quality of life is measured using the EQ-5D-5L [45]. This self-report questionnaire assesses general quality of life using five domains: mobility, self-care, usual activities, pain/discomfort and anxiety/depression. Each dimension has 5 response levels: no problems, slight problems, moderate problems, severe problems and extreme problems. The Dutch norm scores will be used for calculating the mean EQ-5D utility values [95]. The reliability of the EQ-5D-5L is found to be acceptable [45].

The Happiness Question is added as a single question on general happiness in the months prior to the assessment and is scored on a seven-point Likert scale [94]. This scale consists of different states of happiness ranging from completely unhappy to completely happy. Norms for all participating countries are available. For a single happiness item, high test–retest reliability (*r* = 0.86) and good concurrent, convergent and divergent validity have been reported. The Happiness Question [26] has excellent sensitivity to change for patients with borderline PD who were treated with group schema therapy (GST).

Psychosocial functioning and participation is assessed with the World Health Organization Disability Assessment Schedule 2.0 (WHODAS 2.0, [89], a general measure of functioning and disability in major life domains, including understanding and communication, getting around, self-care, getting along with others, life activities and participation in society.

##### Costs

Costs are assessed using a specifically adapted version of the TiC-P [93]. The TiC-P FORCE is a 14-item self-report questionnaire to assess health care costs (part I) and costs resulting from productivity losses (part II) associated with psychiatric disorders. In part I, the number of contacts with different health care providers over the last 6 months is assessed. Part II consists of items regarding absenteeism from paid and unpaid work and presenteeism (i.e. reduced productivity while at work) in the last 6 months.

#### Potential predictors and moderators

Apart from general potential predictors (sociodemographic variables, severity of psychiatric symptoms, type of PD, severity of PD), four specific potential moderators are defined and explained earlier: early childhood trauma, personality organization, autism traits and vulnerable narcissism.

Early childhood trauma is measured by the Childhood Trauma Questionnaire-Short Form (CTQ-SF) [13]. The short form was developed from the original 70-item version [12] and consists of 28 items measuring physical, sexual and emotional abuse and physical and emotional neglect. Reliability and criterion-related validity have been established [13]. A study in the Netherlands confirmed its five-factor model [82].

The Inventory of Personality Organization (IPO-83-NL, [49]) measures personality organization. The IPO-83 is self-report instrument consisting of 83 items on a 5-point Likert scale, based on Kernberg’s structural model of personality organisation [20]. The Dutch version of the IPO has three main scales (Identity Diffusion, Primitive Defence and Reality Testing) and two supplementary scales (Aggression and Moral Values). The IPO-83-NL has good reliability and validity [11].

The Autism-Spectrum Quotient short form (AQ-10) is derived from the original 50-item AQ [3] by a selection of the 10 items with the best discriminant validity. The questionnaire consists of 10 statements with for every statement four response options: strongly agree, slightly, agree, slightly disagree and strongly disagree. At a cut-point of 6, sensitivity was 0.88, specificity was 0.91 and positive predictive value (PPV) was 0.85.

Finally, narcissistic traits are measured with the Dutch Narcissism Scale (Nederlandse Narcisme Schaal, NNS) that consists of 35 items with a 7-point Likert scale, measuring three dimensions of narcissism: overt (‘centrifugal’) narcissism; covert (‘centripetal’) narcissism and isolation [29]. The construction of the covert narcissism subscale is based on the Dutch translation of the hypersensitive narcissism scale [44] and consists of 11 items, with good reliability (Cronbach’s alpha 0.82). Dutch norms are available.

#### Potential mediators

Potentially general working mechanisms across the group therapies, like group cohesion and group climate, are measured by the Dutch translation of the Group Questionnaire (GQ-NL, Van den Heuvel, Klaassen & De Beurs, in prep.). The GQ, developed by Krogel and colleagues [56], is a 30-item self-report measure that assesses the quality of the therapeutic relationship in a group therapy on a 7-point Likert-scale. The GQ consists of three subscales: Positive Bond, Positive Work and Negative Relationship, with a score for each subscale. All three subscales have good reliability. The GQ also assesses relationship structure using three dimensions: member-leader (working alliance), member–member (group cohesion) and member-group (group climate), and it has shown acceptable criterion validity with the Working Alliance Inventory, Group Climate Questionnaire, Therapeutic Factors Inventory and Empathy Scale [85].

In addition, instruments measuring potential working mechanisms related to schema therapy and psychodynamic therapy are administrated in all three treatments. Change in schema modes (schema therapy) is measured by the Schema Mode Inventory 2 (SMI-2)*,* a modified version of the SMI-1 self-report questionnaire [60]. It consists of 143 items on 18 schema modes that are scored on a 6-point Likert scale and measures to what extent dysfunctional as well as functional schema modes are present. Its subscales have satisfactory to high internal consistency (Cronbach’s α ranges from 0.79 to 0.96) and it is considered to be a useful instrument for assessing modes [61]. Newly formulated modes proved to be appropriate for histrionic, avoidant and dependent personality disorder. In line with Yakin [101], the modes Vulnerable Child and Healthy Adult will be analysed a priori and the Avoidant Protector and Impulsive Child modes exploratory. The complete SMI-2 will be administered at baseline, a shortened version with modes that are relevant for Cluster-C PDs is used for the repeated measures.

For psychodynamic therapy, no commonly applied instruments for measuring mediators were identified in the literature. Therefore, we organized an expert meeting with professional experts in psychodynamic therapy. Four potential mediators were selected: insight, experiential avoidance, self-esteem, and defence style. Insight is measured by a subscale of the Client Task-Specific Change Measure-R (CTSC-R), a 16-item client self-report on a 7-point Likert-type scale [97], designed to measure the extent to which clients are able to identify changes, or newly acquired insight associated with particular sessions. A total score on the scale provides an index of client change following the session. The instrument is validated by Watson et al. [98] and showed good psychometric qualities with high internal consistency. Factor analysis showed the instrument comprises two factors, one dominant factor conceptualized as ‘behaviour change’ and a second minor factor conceptualized as ‘awareness and understanding’. This last factor is used as a measurement for insight in the present study. The psychometric properties of the Dutch version are not known.

Experiential avoidance is measured by The Brief Experiential Avoidance Questionnaire (BEAQ), a 15 item self-report measure. It is the shortened version of the 62-item Multidimensional Experiential Avoidance Questionnaire (MEAQ, [40]. Items are scored on a 6-point Likert Scale. A high score reflects a high level of experiential avoidance. Initial validation of the BEAQ has demonstrated good psychometric qualities. The psychometric qualities of the Dutch BEAQ have recently been studied by Slagter et al. A: Measuring experiential avoidance: psychometric properties of the Dutch multidimensional experiential avoidance questionnaire, in preparation.

Self-esteem is assessed with the Rosenberg Self-Esteem Scale (RSES). The RSES is a widely used 10-item Likert scale for measuring self-esteem. Items are answered on a 4-point scale—from strongly agree to strongly disagree—measuring positive and negative feelings towards the self [75]. The Dutch version of the RSES is found to be a one-dimensional scale with high internal consistency and congruent validity and a Cronbach’s alpha of 0.89 [38].

Finally, adaptive or non-adaptive defence style is measured by a short version of the Developmental Profile Inventory-SV (DPI-SV). The DPI is developed to assess psychodynamic personality functioning, based on the frame of reference of the Development Profile (DP). It consists of 9 subscales representing developmental levels of psychodynamic functioning on three domains: Self, Interpersonal Functioning and Defence/coping style [73]. In this study, only the domain defence/coping style is used. Internal consistencies of the subscales were fair to good, ranging: 0.71 to 0.91 in healthy controls and 0.67 to 0.88 in a patient sample. Mean corrected item-total correlations were good, ranging 0.30 to 0.50. Test–retest reliability was good to excellent, with median ICC levels of 0.86 in healthy controls and 0.81 in the patient sample. The DPI also discriminated between patients and healthy controls in a meaningful way.

In addition, for all approaches, after each therapy session, therapists will indicate which interventions they carried out on a 30-items treatment intervention checklist, the G-FORCE Treatment Intervention List (G-FORCE-TIL). This list has been developed by the authors [92], indicating the core interventions per treatment modality on a dichotomous scale (yes/no). Psychometric qualities of this list will be researched.

#### Treatment retention

If the patient discontinues treatment, the therapist fills out a questionnaire about the reasons for dropout. In an exit interview, the patient is motivated by the research assistant to complete the future measurements. If the patient leaves treatment before the end of the protocol and both therapist and the patient regard the treatment as successful, the patient is considered an early completer.

### Participant timeline {13}

An overview of the timeline, interventions and assessments is presented in Table 1.

### Sample size {14}^3^

First, the required sample size was calculated to compare the three types of active treatments. Three pairwise comparisons will be conducted between the active treatment arms (PG vs. SFGT, PG vs. GST; SFGT vs. GST). To detect a medium effect size (*f* = 0.11) in a pairwise comparison of pre-post change between two active treatment arms, with *f*(*v*) = 0.2460 and Cohens *d* = 0.492, with Bonferroni-corrected alpha = 0.0167, a power of 0.80 and within-person correlation coefficient = 0.60, 88 patients are needed per arm. Because three types of therapies will be compared, 3 × 88 = 264 participants are needed to perform three pairwise comparisons. A medium effect size (*f* = 0.11^4^) is chosen to detect a clinically meaningful difference, as opposed to a smaller effect size. The correlation of 0.60 is an educated guess of the correlation between repeated assessments based on the previous RCTs the authors conducted.

The final analysis will be conducted with mixed regression on time series: the change in slope over time between two treatments. Because power analysis for mixed regression is complex and based on complex assumptions about the covariance-structure, a simplified approach was chosen by conducting a power analysis on the pre-post change.

In this study, we must take into account the correlation of observations within group psychotherapy, with an assumed Intra Class Correlation of 0.1. ([88], page 186). Therefore, we will increase the needed numbers with 10%, resulting in a total of 290 participants.

### Recruitment {15}

**Fig. 1:**
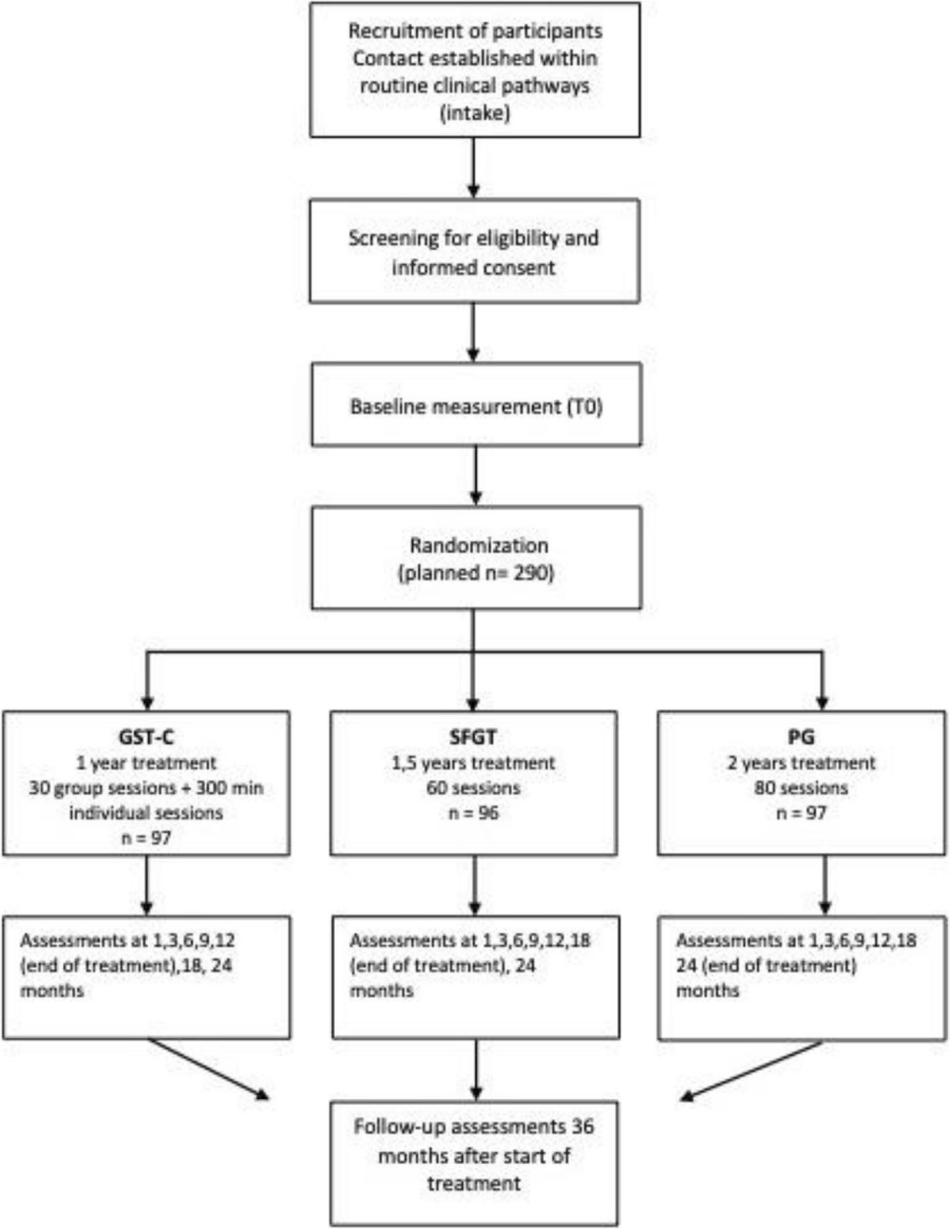
Flow chart of the study design

Patients are recruited from regular referrals to the NPI. As the NPI is part of Arkin, a large Mental Health Institute in Amsterdam, the central enrolment office of Arkin performs a triage based on information of referrers. If needed, additional screening by phone is conducted to select patients suitable for specialized care for personality pathology. Most patients have a history of unsuccessful therapies either for co-morbid disorders or for PD. The intake clinician at the NPI uses the SCID-5-P for obtaining a DSM-5 classification. In case of a diagnosis of a Cluster-C PD (avoidant, dependent or compulsive) or another specified personality disorder with predominantly Cluster-C traits, the patient receives both written and face-to-face information about FORCE and is motivated to participate by the intake clinician. If a patient is interested in participating in the study, a protocolized shared decision procedure is carried out to choose group or individual setting, with group therapy being the first choice. Contraindications for group therapy and/or a strong preference can lead to an indication for individual therapy. In this case, the patient will enrol in I-FORCE (Daniëls et al., in prep.). When a patient prefers to receive group therapy, referral to G-FORCE will follow (see Fig. 1 for an overview of the patient flow).

### Who will take informed consent? {26a}

After referral to G-FORCE, the research assistant will inform the patient and re-check the eligibility. If eligibility is confirmed, the patient will be asked to sign the informed consent. The patient receives all the information about the trial 1 week before the eligibility screening, providing sufficient time for reflection.

### Additional consent provisions for collection and use of participant data and biological specimens {26b}

Not applicable.

## Assignment of interventions: allocation

### Sequence generation {16a}

After informed consent is signed and baseline measurements are completed, the patient is randomized to one of the three treatments. Randomization is stratified by type of Cluster-C PD (avoidant, dependent, obsessive–compulsive, otherwise specified) and location (Amsterdam or Amersfoort/Utrecht) and performed by a computerized randomization program.^5^

### Concealment mechanism {16b}

Allocation sequence is concealed, as randomisation is computer-generated and performed after the patient has signed the informed consent and after baseline assessments are completed.

### Implementation {16c}

The generation of the allocation sequence is done by an independent statistician, who allocates the patient to one of the treatments. The research assistant receives the information on the allocation from the independent statistician and informs the patient about which treatment has been assigned.

## Assignment of interventions: blinding

### Who will be blinded {17a}

Because the study is a clinical psychotherapy trial, it is not possible to blind therapists and participants. However, the research assistants who perform the baseline assessments will be blind for treatment allocation. Also, the other assessments that contain diagnostic interviews are conducted by blinded research assistant. In this way, knowledge about the type of treatment will not affect the outcome of the assessment.

### Procedure for unblinding if needed {17b}

Participants and therapist are not blinded; therefore, there is no procedure for unblinding.

## Data collection and management^6^

### Plans for assessment and collection of outcomes {18a}

Data will be derived from electronic patient records and collected with an electronic Case Report Form (eCRF) using Access. Patients will use an online survey (NetQ) to answer questionnaires. Interviews will be coded and results will be stored in NetQ. Audiotapes will be stored in MS Teams in a folder protected with a password. All data acquired during the study will be anonymized and saved in a study folder on our protected research server. Only the study team has access to this specific study folder.

### Plans to promote participant retention and complete follow-up {18b}

The patients will receive extensive information about the study set-up and requirements during the recruitment. The importance of completion of the follow-up will be stressed. Patients will receive a reimbursement of 20 euros after completing the assessment at 24 months and another 20 euros after completing the last assessment. Patients are allowed to stop at any time during the study and are not obliged to give a reason to discontinue. Questionnaires are completed using an online survey, and therefore patients can do this at any convenient moment, within a time frame of 2 weeks before or after the assessment time points. All patients are reminded throughout the study to fill out the questionnaires. Throughout the follow-up period, the researchers will check responses and if necessary, contact patients for completion of their follow-up.

### Data management {19}

Online Case Report Forms (CRF) are used online (digital platform NetQ), which will allow standardised data capture, as well as facilitate typing, versioning or uploading of documents. In addition, each assessment will have a standardised operational procedure (SOP) to increase internal consistency. Such SOPs will determine who may conduct the assessment (either online or face-to-face), evaluation steps and standardised communication with research participants.

### Confidentiality {27}

Research data will be stored using a study identification code for each participant. The key document will only be accessible to the principal investigators and the statistician of the mental health institute (Arkin) and will be safeguarded by the principal investigator according to research guidelines after completion of the study. Publications will only report aggregated statistical information, which cannot be related to individual participants.

### Plans for collection, laboratory evaluation and storage of biological specimens for genetic or molecular analysis in this trial/future use {33}

Not applicable.

## Statistical methods^7^

### Statistical methods for primary and secondary outcomes {20a}

#### Primary study parameter(s)

##### Treatment response: ADP-IV

All analyses will be conducted according to the intention-to-treat principle. Primary and secondary outcomes are analysed with Linear Mixed Models, with random effect of group if estimation allows. The primary outcome measure is the change in total score on the ADP-IV at 24 months using dimensional scoring algorithms [77]. Change in the primary outcome measure and the relative effectiveness of the three treatments will be analysed using mixed regression so that all available data are used, taking into account the levels of participant, time and group. The underlying distribution of the mixed regression model will be determined based on the distribution of residuals (e.g. normal, gamma, negative binomial). Per-protocol analyses will also be conducted to test for robustness.

Because of the dosage differences, an additional comparison of effectiveness will be conducted at the different end points of the treatments (resp. 12, 18 and 24 months).

##### Differential treatment response

To gain more insight into differential treatment response, we will first examine which of the potential predictors actually predict (differential) treatment response. In the moderator analyses, we will utilize the Personalized Advantage Index (PAI) analysis developed by DeRubeis [24], previously used in the research group of the principal investigator [15, 16]. In step 1, four univariate regression models will be built for each specific predictor, with the predictor and separate variables for the interaction with the three interventions (moderators). In step 2, a multivariable regression model with the significant predictors (and their interaction with treatment) from step 1 will be developed, to examine the relative contribution of each potential predictor or moderator. The outcome variable is the ADP-IV. In addition, we will make use of a data-driven approach, in which machine learning techniques are used to select the moderators to be added into the PAI algorithm.

##### Mechanisms of change

It is hypothesized that the interventions exert a positive effect on the severity of Cluster-C manifestations measured by the ADP-IV, through their impact on the underlying general and theory specific mechanisms of change. To identify non-specific and specific mechanisms of change and the strength of the factors involved, both multilevel mediation models and structural equation models will be used. These analyses are based on the Latent Change Score models for mediation used in the research group of the principal investigator [15, 16].

#### Secondary study parameters

##### (Differential) treatment response: secondary study parameters

Mixed regression analysis is used to examine change on the secondary outcome measures in the three treatments. The secondary outcome measures include severity of cluster C PD, reliable change and recovery, personality functioning, general functioning, general psychopathology, quality of life and happiness. As the PD-severity indices AVPDSI, DPDSI and OCPDS vary per primary PD, they will be first standardized before they are analysed in the complete sample. This analysis will be controlled for primary PD, to account for possible differences in sensitivity to change. The underlying distribution of the mixed regression model will be determined based on the variable type (scale, nominal) and the distribution of residuals (e.g. normal, gamma, negative binomial). In addition, preliminary analyses will test differential treatment retention using survival analysis and mixed logistic regression. Medication confounds will be examined.

##### Cost-effectiveness

The cost-effectiveness analysis (CEA) will be conducted from a societal perspective. Within the CEA, the difference in societal costs (measured by the TiC-P FORCE at baseline and after 6, 12, 18, 24 and 36 months) generated by patients in the three conditions will be related to the difference in clinical effects (measured with the ADP-IV and quality-adjusted life-years (QALYs) based on the EQ-5D-5L) over the course of 36 months. Missing cost and effect data will be imputed using multiple imputation. Mixed model regression analyses will be used to estimate cost and effect differences between groups. Bootstrapping with 5000 replications will be used to estimate 95% confidence intervals around cost differences and the uncertainty surrounding the incremental cost-effectiveness ratios (ICERs). Uncertainty surrounding the ICERs will be graphically presented on cost-effectiveness planes. Cost-effectiveness acceptability curves [32] will also be estimated. Adjustment for confounders and effect modifiers will be done if necessary.

#### Interim analyses {21b}

No interim analyses are planned.

#### Methods for additional analyses (e.g. subgroup analyses) {20b}

Additionally, subgroup analyses are planned for the three primary cluster C PDs (avoidant, dependent, obsessive–compulsive) and the otherwise specified PD with predominantly cluster C traits.

#### Methods in analysis to handle protocol non-adherence and any statistical methods to handle missing data {20c}

An intention-to-treat analysis will be performed for the primary outcome measure. Missing data will be reduced to a minimum by using suitable measures described above.

#### Plans to give access to the full protocol, participant level-data and statistical code {31c}

On request and with the agreement of the research group, the full protocol can be made available by the corresponding author. The data on participant level will not be shared with external parties or on external repositories, because of privacy issues, sensitivity of the data and the policies for data security.

## Oversight and monitoring

### Composition of the coordinating centre and trial steering committee {5d}

This study is designed, performed and coordinated at Arkin/NPI. Daily support for the trial is carried out by as follows:

- Principal investigator: takes supervision of the trial and medical responsibility of the patients.
- Data manager: organizes data capture, safeguards quality and data.
- Study coordinator: trial registration, coordinates the study, annual safety reports.
- Research assistant: identifies potential recruits, takes informed consent, conducts interviews and ensures follow-up according to protocol.

The research committee meets biweekly. There is no trial steering committee or stakeholder and public involvement group. Contact with the client advice board from Arkin is established.

### Composition of the data monitoring committee, its role and reporting structure {21a}

A DSMB has not been assigned for this study in accordance with the ethical committee of the VU Amsterdam.

### Adverse event reporting and harms {22}

For participants of this study, no direct risks are expected or foreseen. Any adverse events reported by the patient or observed by the investigator, other members of the research team or therapists will be documented. An adverse event is defined as an undesirable experience that happens to a participant during the study, whether or not related to the experimental intervention.

### Frequency and plans for auditing trial conduct {23}

At the moment, no audit is planned for trial conduct.

### Plans for communicating important protocol amendments to relevant parties (e.g. trial participants, ethical committees) {25}

Important protocol amendments will be proposed to the ethical committee of the VU Amsterdam and if applicable, with the therapists and the research participants.

### Dissemination plans {31a}

We will submit the trial results to a peer-reviewed journal. The final article will be shared with all the participants and therapists of the study.

## Discussion

This randomized controlled trial aims to bridge the gap between actual clinical practice that offers different types of group therapy for Cluster-C PDs and the almost absence of an evidence base. This scientific neglect in the study of Cluster-C PDs makes it practically impossible to inform patients reliably about valuable treatment options. Considering the high frequency of Cluster-C PDs and its negative impact on quality of life and the effectiveness of treatments for other common mental health disorders, there is clearly an urgent need to ameliorate this state of affairs.

With individual therapy as the focus of previous research [9, 83], G-FORCE will be the first RCT on group therapy for Cluster-C PDs. Group therapy has many advantages, such as the possibility of interpersonal learning. Also, several meta‐analyses have found no difference between effectiveness of group and individual therapy for several theoretical orientations and across various disorders, such as anxiety and mood disorders [17, 76].

G-FORCE is designed to investigate the differential effectiveness of two forms of schema group therapy (GST-C and SFGT) and psychodynamic group therapy for Cluster-C PDs and to study the general and theory-specific predictors and working mechanisms. In this study, patients are not only included with a specific Cluster-C PD but also with a diagnosis of an otherwise specified PD with Cluster-C traits and with a wide range of psychiatric comorbidity.

### Strengths

To our best knowledge, this study is the first to compare directly two important theoretical frameworks of group therapy: schema group psychotherapy and psychodynamic group psychotherapy. Regarding schema group therapy, controlled studies do exist [31, 100], but all were focused on borderline PD (BPD). With the development of a group schema therapy for Cluster-C patients (GST-C), the first promising results of a pilot study on its effectiveness, and a currently running RCT comparing GST-C with individual schema therapy [7, 43], the evidence for schema therapy for this type of patients is starting to grow. G-FORCE contributes to this line of research, with a unique comparison between two forms of schema group therapy and psychodynamic group therapy.

Another distinctive feature of the study is that the three forms of group therapy are dimensionally distributed on a spectrum, with structured, short duration GST-C on the one end, unstructured and long duration PG on the other end, and SFGT in the middle, representing a combination of structured group schema therapy and unstructured dynamic group therapy.

In the field of PG, evidence is mainly coming from open cohorts and mixed diagnostic groups. This shortcoming of evidence also reflects a lack of uniformed and sufficiently transparent manuals on psychodynamic or psycho-analytic group therapy [76]. By designing the G-FORCE study, a protocol was developed for PG, meeting the need for more transparency that is asked by patients and other stakeholders.

The only controlled study on the effectiveness for PDs compared short-term with long-term psychoanalytic group therapy [63]. With our study, PG will be compared with schema therapy, thus broadening the evidence to the differential effectiveness of group therapy approaches stemming from distinctive theoretical frameworks.

Another important strength of this trial is its pragmatic character. In pragmatic trials, the interventions in real-life routine practice settings are researched [34]. This means the findings are highly generalizable and potentially beneficial for clinical practice [71]. The majority of the participants of this study received individual treatment before and will receive group therapy for the first time.

Finally, the design of the current study enables us to investigate both the predictors and treatment processes during group therapy. Treatment interventions, potential mediators and outcome are measured repeatedly during treatment, meeting the condition of establishing a temporal relationship between the variables. In this way, we aim to contribute to answer the question which treatment works for whom and why.

### Limitations

Some limitations must be considered. First, we are not able to compare any of the treatments with a ‘golden standard’. Also, no comparison will be made with a non-active control-group as this is perceived unethical in view of the long waiting lists for the participating departments and the long duration of the therapies. Moreover, all the interventions in this study could possibly be considered as the treatment as usual (TAU) in Dutch clinical practice or as experimental interventions that have yet to be established. Compared to PG, GST-C is more recently developed and based on a clearly defined protocol. For SFGT, a protocol had been published internally, based on publication of Aalders & van Dijk [1]. PG has, by nature, a more open structure and more differences in how it is applied in clinical practice. That is why we have developed a manual for the psychodynamic group with experts in the field (Van Dam, El Boushy, Meijers & Van den Heuvel, 2021, internal publication), enabling this therapy to be delivered in a transparent and uniform way. A more general description has been published recently as well [84]. This protocol enables us to assess adherence and competence also of PG. We therefore consider none of the applied therapy forms as a condition that would represent TAU. In addition, all therapies are offered in a highly specialized institute for PD. This implies an ongoing context of peer—and expert supervision and quality evaluations that are normally not available in context of TAU, while the main therapists are all highly qualified, with broad experience in PG.

In the current study, no comparison is made between group therapy and individual therapy. Although this would be valuable, we expected a negative impact of randomisation between individual and group therapy, such as increased dropout rates and difficulties with motivation and enrolment of patients. Therefore, a separate RCT on individual therapy for cluster C pathology (I-FORCE) has been developed and is currently running (Daniëls et al. in prep.).

Another limitation is the different duration of the treatment modalities. Although we ruled out the confounding effect of time by setting the end point of the main outcome measurements at 24 months, it will not be clear what the confounding effect of dosage could be. Difference in dosage is due to the pragmatic character of the study in researching existing group therapy formats, with varying duration and dosage. Because of these different dosages, determining cost-effectiveness at follow-up is of extra importance in this study. However, the difference in dosage and amount of structure in the compared therapies gives, in combination with the extensive study of predictors and potential moderators, the opportunity to gain valuable knowledge about which therapy is best for which type of Cluster C patient, hereby optimizing the possibility of matched or personalized care.

To conclude, in the sample size calculation, we did not adjust for dropout, which could negatively affect the power. Nevertheless, mixed regression with multiple measurements over 2 years is expected to balance out this potential loss of power because of the particular strength of this type of analysis.

As far as we know, G-FORCE will be the first large pragmatic RCT on the effectiveness of group therapy for Cluster-C PDs, contributing to growing evidence on effective Cluster-C treatments and group psychotherapy. With its focus on possible predictors and mediators, G-FORCE will increase our understanding on which therapy works for whom and why, helping to recommend the best available clinical treatment for the individual patient with a Cluster-C PD diagnosis.

## Trial status

Recruitment has started on October 1, 2020. Recruitment will be completed on approximately October 1, 2023. At the time of submission of the current protocol, a total of 167 patients is already included in the study.

## Data Availability

According to the Dutch General Data Protection Regulation, the datasets used and/or analysed during this trial cannot be provided to external parties, neither will the data be published on a public repository.

## Abbreviations

ADP: Assessment of DSM-IV Personality Disorders questionnaire
AE: Adverse event
AQ: Autism Questionnaire
AVPDSI: Avoidant personality disorder severity index
BEAQ: Brief Experiential Avoidance Questionnaire
BSI: Brief Symptom Inventory
CCMO: Central Committee on Research Involving Human Subjects; in Dutch: Centrale Commissie Mensgebonden Onderzoek
DEPDSI: Dependent Personality Disorder Severity Index
SMB: Data Safety Monitoring Board
CTSC-R: Client Task-Specific Change Measure-Revised
CTQ-SF: Childhood Trauma Questionnaire-Short Form
DPI-SV: Developmental Profile Inventory-Short Version
G-FORCE: Group Psychotherapy FOR Cluster-C Personality Disorders Effect Study
G-FORCE-TIL: G-FORCE Treatment Intervention List
GST: Group schema therapy
GQ: Group Questionnaire
ICER: Incremental cost-effectiveness ratio
IPO: The Inventory of Personality Organization
NNS: Nederlandse Narcisme Schaal
OCPDSI: Compulsive Personality Disorder Severity Index
PD: Personality disorder
PG: Psychodynamic group therapy
RCT: Randomized controlled trial
RSES: Rosenberg Self Esteem Scale
(S)AE: Serious adverse event
SCID-II: Structured Clinical Interview for DSM-IV Axis-II Disorders
SCID-5-P: Structured Clinical Interview for DSM-5 Personality Disorders
SMI: Schema Mode Inventory
SFGT: Schema-focused group therapy
TAU: Treatment as usual
TiC-P: Treatment Inventory Cost in Psychiatric Patients
WHODAS: World Health Organization Disability Assessment Schedule

## Footnotes

1 The in- and exclusion criteria are similar to the criteria described in the protocol article of the I-FORCE study [22].

2 Due to equal measurements in I-FORCE and G-FORCE, the description of the measurements is partially identical in both manuscripts [22].

3 The description of the calculation of the sample size is identical to the description in the I-FORCE protocol paper [22].

4 This effect size *f* is used in GPower for the within-between interaction and is not the equivalent of the between effect size *f*. We therefore reported the equivalent effect sizes according to Cohen (i.e., *f*(*v*) = .2460) and Cohen’s *d* = .492—which are approximately medium.

5 We changed the procedure of randomization during of the process of inclusion. Earlier, randomization was performed before the baseline assessments. Any potential effect of this change in procedure will be monitored and investigated.

6 The data collection and management for G-FORCE uses the same procedures as the I-FORCE study. Therefore, the description in this article is similar to the I-FORCE protocol article [22].

7 The statistical methods of G-FORCE are similar in design and procedure to I-FORCE. Therefore, the description of the statistical methods is highly identical to the description in the I-FORCE protocol paper [22].
